# Why the Epistemic Objection Against Using Sentience as Criterion of Moral Status is Flawed

**DOI:** 10.1007/s11948-022-00408-y

**Published:** 2022-10-28

**Authors:** Leonard Dung

**Affiliations:** Institute for Philosophy II, Ruhr-University Bochum, 44801 Bochum, Germany

**Keywords:** Sentience, Machine consciousness, Moral status, Robot rights, Animal ethics, Uncertainty

## Abstract

According to a common view, sentience is necessary and sufficient for moral status. In other words, whether a being has intrinsic moral relevance is determined by its capacity for conscious experience. The *epistemic objection* derives from our profound uncertainty about sentience. According to this objection, we cannot use sentience as a *criterion* to ascribe moral status in practice because we won’t know in the foreseeable future which animals and AI systems are sentient while ethical questions regarding the possession of moral status are urgent. Therefore, we need to formulate an alternative criterion. I argue that the epistemic objection is dissolved once one clearly distinguishes between the question what determines moral status and what criterion should be employed in practice to ascribe moral status. Epistemic concerns are irrelevant to the former question and—I will argue—criteria of moral status have inescapably to be based on sentience, if one concedes that sentience determines moral status. It follows that doubts about our epistemic access to sentience cannot be used to motivate an alternative criterion of moral status. If sentience turns out to be unknowable, then moral status is unknowable. However, I briefly advocate against such strong pessimism.

## Introduction

Our everyday life is entangled in various ways with the fate of non-human animals and artificial intelligences (AI). We keep animals as pets, consume food and other products made from animals and encounter many of them during the course of our day. AI systems continue to intrude deeper into our lives, for instance as personal assistants (like Amazon’s Alexa) or as recommender systems used by social media platforms. Moreover, AI rapidly improves in computational power and problem-solving ability. Since human actions impact animals as well as AI in multi-facetted and far-reaching ways, it is important to investigate what we owe to these non-human beings. While the modern animal rights movement dates back at least to Singer’s “Animal Liberation” (Singer, 1977), a robots right movement is in the process of emerging (Gunkel, 2018; Schwitzgebel & Garza, 2015; Ziesche & Yampolskiy, 2018).

This paper will be broadly about what it takes for beings—like animals and AI—to have moral status, i.e., to matter morally for their own sake. More precisely, I will defend the claim that sentience, i.e., the capacity to have conscious experiences, should be used as criterion for attributing moral status^1^ (Jaworska & Tannenbaum, 2021) from the *epistemic objection*. According to this objection, measuring the distribution of sentience is intractable, at least for the time being. However, we urgently need to assign moral status to guide our interactions with non-human beings. Therefore, we should use a criterion for moral status other than sentience, e.g., the possession of desires or psychological equivalence to generally acknowledged moral patients (e.g., Danaher, 2020; Shevlin, 2021). I will claim that the epistemic objection is fallacious. Even if sentience turns out to be completely beyond the scope of scientific investigation, there can be no alternative criterion for ascribing moral status. In this case, we would doomed to stay ignorant regarding the moral status of many animals and machines. That being said, we do not need to be quite so pessimistic regarding the prospects for scientific knowledge about the distribution of sentience.

In the next section, I will properly introduce the key terms necessary to frame this debate. In particular, I will introduce the distinction between grounds and criteria of moral status on which the later argument relies and describe the view that sentience serves as a proper criterion for moral status. In section “The Epistemic Objection”, I explain and motivate the epistemic objection to the claim that sentience should serve as a criterion for moral status. In section “Criteria for Moral Status and Grounds of Moral Status”, I refute the epistemic objection. Subsequently, I outline how we can successfully use sentience as a criterion of moral status while acknowledging the uncertainty of sentience attributions. Section “Conclusion” concludes.

## Sentience and Psychological Moral Patiency

I will now elucidate the notions ‘moral status’ and ‘sentience’. A being possesses moral status if and only if it matters morally for its own sake (Jaworska & Tannenbaum, 2021). That is, if a being has moral status, we have obligations to that being in virtue of its intrinsic properties, not because it matters to someone else.^2^ We ought to consider how our decisions affect beings with moral status in our moral deliberations. Shevlin (2021) helpfully distinguishes the concept of *psychological moral patiency* (PMP) from the notion of moral status generally. The former is “a form of moral status that may arise in virtue of a possession of specific psychological capacities such as sentience, autonomy, desires and so on” (p. 460). That is, PMP is a kind of moral status which is special in that it obtains in virtue of psychological properties.^3^

Like Shevlin, I remain neutral on the question whether there is a form of moral status that does not presuppose the possession of any psychological features.^4^ Beings might have moral status even if they are not psychological moral patients. What matters to the project of this paper is that *some* beings possess a *form* of moral status which is grounded in their psychological capacities. This seems to be the case since we attribute moral status to beings with a complex and multi-facetted mind like, for instance, humans or chimpanzees. Furthermore, we treat non-mental entities usually as morally less important, if at all.

According to a standard view, moral status is determined by sentience (Kriegel, 2019; Nussbaum, 2007; Schukraft, 2020; Singer, 2011).^5^ The claim that PMP is determined by sentience is implied by the claim that moral status is determined by sentience. Thus, the former is equally or even more plausible than the latter. I take *sentience* to be the capacity to have phenomenally conscious experiences. This requires two clarifications. First, an experience is phenomenally conscious if and only if there is something “it is like” (Nagel, 1974) to undergo the experience. Phenomenally conscious experiences are felt subjectively, from the first-person point of view. Second, many people understand sentience to be the capacity to have conscious experiences with a *valence*, i.e., experiences that feel good or bad, like pain, fear, joy or relief (Birch et al., 2021). Consequently, many think that only valenced experience is relevant to moral status.^6^

As a terminological stipulation, I understand sentience as the capacity for conscious experience in general. I take *sentientism* to be the view that PMP is determined by sentience, i.e., beings are psychological moral patients in virtue of being sentient. Sentientism is supposed to be neutral on the question whether non-valenced experiences are relevant to moral status. It implies the following two claims:

1. Sentience is necessary for PMP.
2. The capacity for *valenced* conscious experience is sufficient for PMP.

I choose this combination of claims because it is the least demanding version of the view that sentience is necessary and sufficient for PMP.

It is hard to come up with direct arguments for sentientism, since the view rests mainly on a deep-seated intuition, not theoretical argument. However, at the very least, if a creature feels bad, this always seems to create a moral reason to help it.^7^ This suggests that there is a form of moral status for which sentience suffices. Since many philosophers seem to accept the force of the intuitions and arguments for sentientism, I will take them for granted here. The main purpose of this paper is to *defend* the use of sentience as a criterion for ascriptions of PMP against a particular objection, not to argue for sentientism.

Sentientism is a claim about what *grounds* PMP. It concerns the set of facts which ontologically determines PMP. This contrasts with *criteria* of PMP. I call a feature which is adequate (in practice) to assess which beings are psychological moral patients a *criterion* of PMP. A criterion is a feature which can in actual situations be used to identify psychological moral patients. For illustration, one may say that water’s chemical composition, H2O, is the *ground* of water, while the property of being a tasteless, odorless and transparent liquid serves as *criterion* for identifying water in many circumstances. My main argument will rest on the distinction between grounds and criteria.

Sentientism seems to supply a straightforward criterion: According to sentientism, when we need to know whether a being has PMP, we should test whether it is sentient. According to this criterion, we should resort to the flourishing research programs on animal (Birch et al., 2020; Sneddon et al., 2014) and artificial consciousness (Dehaene et al., 2017; Elamrani & Yampolskiy, 2019; Tononi & Koch, 2015) when we are in doubt regarding the moral status of animals or AI.

In the following section, I will outline the main objection to regarding sentience as criterion of PMP. According to this epistemic objection, sentience cannot serve as criterion of PMP because, for many animals and AI systems, there is no reliable way of determining whether they are sentient. In the subsequent section, I will address the relation of this criticism of the use of sentience as a criterion for assessing PMP to sentientism as a view of the ground of PMP.

## The Epistemic Objection

In a nutshell, the epistemic objection is based on the difficulty of discerning which beings are sentient. In respect to animals which are evolutionarily distant from humans, e.g. invertebrates like octopodes or insects, we cannot trust analogies to human behavior and neurophysiology to detect conscious experience. This difficulty is even more pronounced in the case of AI, whose genesis and physical organization is even more removed from humans. Pessimism about our ability to ascertain the distribution of sentience is borne out by the fact that researchers vehemently disagree on which animals are sentient. Views range from a restriction of consciousness to humans and perhaps apes (Carruthers, 1998; Dennett, 1995) over including many invertebrates (Barron & Klein, 2016; Godfrey-Smith, 2020) to attributing sentience to almost any or literally every entity (Goff, 2017; Tononi & Koch, 2015). Since these disagreements involve foundational metaphysical (Schwitzgebel, 2020) and methodological (Irvine, 2012) controversies, they probably will not be resolved by new empirical data any time soon.

While questions of the distribution of sentience are shrouded in uncertainty and disagreement, the need for an account of the distribution of PMP is pressing. There are many animals which may be subject to grave harm and injustice, in case they are moral patients. Furthermore, without an account of PMP, we don’t know how close we are to designing AI which possesses moral rights. The two assumptions that we need to be able to attribute PMP now and that we (momentarily) cannot reliably ascertain which beings are sentient constitute the core of the epistemic objection. If we cannot reliably detect sentience, then we cannot know which beings are psychological moral patients. Thus, we cannot use sentience as a criterion for PMP in practice. Given that there is an urgent practical need for this knowledge, we need to resort to a different criterion of PMP for the time being.

Many authors use concerns about the epistemic inaccessibility of sentience to motivate alternative criteria of PMP. Shevlin (2021) claims that the most difficult challenge sentience-based approaches to moral patiency face is the question of “how we can ever establish whether a given system is conscious” (p. 464). Shevlin (2020a) interprets the same worry as a key weakness of the view that sentience should be used as criterion for PMP. Gunkel (2019) regards the problem of determining which AI systems are conscious as one of the main reasons to refrain from trying to ascribe moral status based on sentience. Finally, Dawkins (2017)—characterizing the investigation of animal consciousness as “the most difficult of all biological problems” (p. 4)—claims that animal welfare science should proceed independently of questions of animal consciousness. In light of the connection between ascriptions of welfare and of PMP, this can be seen as an implicit instance of the epistemic objection.^8^

Based on these epistemic concerns, all these authors propose competing criteria of PMP.^9^ At least partially, these criteria are motivated by their putative higher amenability to scientific investigation. Shevlin (2021, p. 466) proposes a “cognitive equivalence strategy” according to which attributions of PMP should depend on the extent to which the being in question shares psychological capacities with beings which we consider to be moral patients. Since many psychological capacities are arguably easier to examine than sentience, this criterion makes attributions of PMP possible in practice.

According to Gunkel (2019), attributions of PMP do not depend on intrinsic properties of the putative moral patient at all. Instead, moral status should be conferred according to our objectively observable relationships to the being in question and our responses to and interactions with it.

Dawkins (2008, 2012, 2021) pioneered and advocated for an approach to animal welfare which in essence argues (roughly) that animal wellbeing should mainly be understood in terms of two components: health and the satisfaction of the animals’ preferences. Health can be tested physiologically and preferences manifest themselves in behavior (Dawkins, 2021). Hence, health and preference satisfaction are both “objectively measurable. Neither needs any necessary involvement with conscious experiences, although both leave open the possibility that these may be present” (Dawkins, 2017, p. 6).^10^

To summarize, the epistemic objection consists in using our lack of knowledge of sentience as a reason to reject sentience as criterion for attributing PMP. Adherents of the epistemic objection propose different criteria for PMP. While their views differ markedly from each other, they are united in the ambition to develop a criterion which is more open to empirical investigation than sentience. In the next section, I will present a dilemma for proponents of the epistemic argument. I will set aside the question of whether finding out the distribution of sentience is indeed as unrealistic for current science as proponents of the epistemic objection suppose. Instead, according to my counterargument, whether we should investigate PMP by investigating sentience is independent of the epistemic accessibility of sentience.

## Criteria for Moral Status and Grounds of Moral Status

My counterargument to the epistemic objection rests on the crucial distinction between *grounds* and *criteria* of moral status which I described in section “Sentience and Psychological Moral Patiency”. Sentientism is the claim that a being is a psychological moral patient in virtue of being sentient. It concerns the grounds of PMP. Sentientism is thus a metaphysical claim which is motivated, criticized and defended via the methods proprietary to normative ethics.

By contrast, a criterion of PMP is used *in practice* to ascribe PMP. For this reason, such a criterion is sensitive to pragmatic and epistemic factors. It must—given actual constraints of time and other resources—be possible to ascertain whether a being satisfies the criterion or not. Otherwise, the criterion is useless. Phrased in terms of disciplinary boundaries, questions about the criterion of PMP belong to applied ethics.

Most proponents of the epistemic objection don’t explicitly appeal to the distinction between grounds and criteria of PMP. However, the distinction is (employing other concepts) mentioned by Danaher (2020). His view, *ethical behaviorism*, claims that observable behavioral relations and reactions of other entities can provide *sufficient warrant* for believing that they have rights against us. He regards this epistemic claim as an application of methodological behaviorism and contrasts it with ontological behaviorism to which he is not committed. For ethical behaviorists need neither “deny the existence of inner mental states, nor deny that those inner mental states provide the ultimate metaphysical ground for our ethical principles” (ibid.). He says that “they can agree that sentience provides the ultimate metaphysical warrant for our duties to animals and humans.” (ibid). In short, Danaher allows that sentience may be the ground of moral status, yet he argues that the criterion of moral status is rough behavioral equivalency to other entities which we take to have moral status. He holds this criterion to be superior because “it respects our epistemic limits” (ibid.).

To summarize, Danaher argues that we need a criterion of PMP other than sentience because sentience may not be sufficiently epistemically accessible. Hence, Danaher presents another instance of the epistemic objection. At the same time, his distinction between ethical and ontological behaviorism mirrors the distinction between criteria and grounds of moral status. For this reason, it helps to uncover the general flaw underlying the epistemic objection, as we will see shortly.

To be clear, my subsequent argument does not directly reject the specific criteria of PMP suggested by Shevlin, Dawkins, Gunkel and Danaher. For the criteria they propose may be adequate despite my argument, either if sentientism is false (such that PMP can be grounded in properties other than sentience) or if the criteria they propose actually reliably track sentience. However, in any case, concerns about our capacity to know the distribution of sentience don’t support those criteria. This is because these epistemic concerns are not relevant to sentientism and are inconsistent with arguing that the criteria suggested reliably track sentience. Thus, the epistemic objection is fallacious. I will now present my argument in detail.

The distinction between grounds and criteria of moral status reveals that there are two types of objections against the claim that sentience serves as criterion of PMP. First, one may simply claim that sentientism is false, i.e., sentience is not the ground of PMP. If sentientism is false, then—in the absence of further argument—we have no reason to use sentience as criterion of PMP.^11^ Even if we would identify which beings are sentient, the ethical relevance of this discovery would be questionable. Second, one may hold that sentientism is true but that sentience is nevertheless not the criterion of PMP. This is consistent with the view endorsed by Danaher. Sentience may be the ground but not the criterion of PMP.

The distinction between those two types of objections against sentience as a criterion of PMP is exhaustive since sentientism has to be either true or false. In what follows, I will argue that the epistemic objection neither supports the contention that sentientism is false nor the thesis that, if sentientism is true, sentience is nevertheless not the criterion for ascribing PMP. Since these two types of objections to sentience as a criterion are exhaustive and the epistemic objection cannot support either one of them, the epistemic objection cannot support the claim that sentience is not the criterion of PMP.

Let us first discuss the first type of objection. That is, does the epistemic objection show that sentientism is false? It seems clear that it does not. Sentientism is a metaphysical claim about the grounds of PMP. Its truth depends on what PMP ultimately consists in. Whether or not sentientism is true is examined with methods of normative ethics. For this investigation, a paradigmatic methodology is the following: One envisages a scenario which includes beings or states without consciousness and contrasts it with a scenario which is almost identical except that it involves consciousness to find out how the difference in consciousness influences intuitions about moral weight (e.g., Kriegel, 2019; Levy, 2014). The outcome of this procedure is independent from the epistemic accessibility of sentience.

More generally, the claim that sentience grounds PMP does not imply, or even suggest, that we can reliably test for sentience in most beings. For it is perfectly coherent, although it would be unfortunate, that facts about PMP are unknowable to us. To illustrate this, consider that water might be said to be grounded in its chemical structure. Even if most objects—including instances of water—could not be tested for their chemical structure, this does not refute the contention that water is determined by H2O. Analogously, even if the distribution of sentience cannot be known, this is compatible with the claim that sentience determines PMP.^12^ To conclude the rejection of the first interpretation of the epistemic objection: Since sentientism is consistent with the unknowability of sentience in non-human animals and machines, the epistemic objection does not threaten sentientism.

Let’s move on to the second interpretation of the epistemic objection. Does the epistemic objection provide reason to believe that, even if sentientism is true, sentience is not the criterion of PMP? It does not. The irrelevance of epistemic considerations to the question of whether sentience is the proper criterion for PMP can be demonstrated through a dilemma objection. *Ex hypothesi*, facts about sentience determine facts about PMP. Suppose someone proposes a criterion of PMP according to which the detection of property F should be taken as providing sufficient warrant for ascribing PMP.^13^ To overcome the epistemic objection against sentience as a criterion, F needs to be a property that we can reliably detect.

Tautologically, F either reliably tracks the presence of sentience or it does not reliably track the presence of sentience. If F reliably tracks sentience, then F can be used as a measure of sentience. In this case, the epistemic objection rests on the false premise that sentience cannot be measured. By relying on F as an indicator of sentience, we can measure the distribution of sentience and thus assign PMP accordingly. Given this horn of the dilemma, F serves as a criterion of PMP by being an indicator of sentience, contrary to the conclusion of the epistemic objection.

According to the other horn of the dilemma, F does not reliably track sentience. If F does not correlate with sentience but sentience is necessary and sufficient for PMP (remember that this part of the argument presupposes sentientism), then F does not correlate with PMP. Hence, if F does not track sentience, then F does not track PMP. Consequently, F cannot be used to infer PMP and thus not serve as a criterion of PMP. By joining the conclusions of both horns of the dilemma, we see that, given sentientism, any feature F either serves as criterion of PMP by being a reliable indicator of sentience or does not track PMP and thus cannot be used as a criterion for it. We can conclude that, given sentientism, any putative criterion of PMP either is an indicator of sentience or fails since it does not in fact indicate PMP. Given sentientism and the assumption of the epistemic objection that there are no reliable indicators of sentience, it follows that there is no criterion for PMP.

The water analogy illustrates this inseparability of ground and criteria of PMP. Given that the chemical structure H2O grounds the property of being water, superficial features of water like its lack of taste, odor and color can indicate the presence of water—and thus serve as its criterion—only insofar as they indicate the presence of H2O. It would make no sense to propose an alternative criterion of water which does not presuppose our ability to detect H2O, if we grant that being H2O is necessary and sufficient for being water. However, this is structurally analogous to what proponents of the epistemic objection do, if they grant that sentience grounds PMP.

We have arrived at the following results. As soon as we accept sentientism, we are committed to the view that an investigation of the distribution of PMP equals an investigation of the distribution of sentience. One may still propose different indicators of PMP, but those are only relevant insofar as they shed light on the distribution of sentience. They can have no independent relevance for PMP. It follows that pessimism regarding our prospects for discovering the distribution of consciousness is irrelevant to the choice of a criterion of PMP, if sentientism is true. On the one hand, if one can propose useful indicators of sentience, this pessimism is unjustified. On the other, if there are no useful indicators of sentience, then there is no criterion of PMP.

Either way, pessimism regarding the investigation of animal sentience does not concern criteria of PMP, if sentientism is true. Since pessimism regarding the investigation of animal sentience does not constitute an argument against sentientism, it is altogether irrelevant to choosing a proper criterion for ascribing PMP. If sentientism is true but animal and AI sentience turns out to be beyond the reach of science, the rational response is not to modify the criterion of PMP but rather to despair.^14^

It is crucial that the argument I presented does not depend on specific properties of sentience. It generalizes to every property which is thought to be the ground of PMP. Whenever we have strong reason to believe that a property G in fact grounds PMP, the preceding discussion demonstrates that G should be employed as criterion for assigning PMP, no matter how grave the obstacles in figuring out the distribution of G are. From my discussion emerges the *autonomy of normative ethics*. The normative ethical project of gaining knowledge about the ground of PMP should unfold independently of concerns about how this knowledge can be used. Pragmatic scruples regarding the applicability of insights about the grounds of PMP are irrelevant. They affect neither this foundational normative discussion nor the choice of criteria for ascribing PMP.

I have presented the main argument of this paper. In the next section, I will make some suggestions for using sentience as criterion of PMP, given that we are uncertain about which actual and possible beings are sentient. In doing that, I reject the pessimistic view according to which we cannot know how to act in relation to many non-human beings, since their PMP is unclear. Importantly, the preceding argument to the effect that there can be no criterion of PMP other than sentience does not depend on the moderately optimistic outlook I develop next section.

## Ethics and Uncertainty of Sentience

The worry we now confront is the following: Assuming sentientism, the preceding argument showed that we have no alternative to employing sentience as criterion of PMP. If proponents of the epistemic objection are correct in holding that we won’t know which animals and AI systems are sentient in the foreseeable future, we have no non-arbitrary way of assigning PMP. Hence, as far as some courses of action open to us concern these beings, we cannot know what our moral obligations are.

I don’t think this worry is *entirely* misplaced. However, I am significantly less pessimistic for two reasons: First, I do not think that there exist many beings where we are completely ignorant regarding their conscious experience. Second, there are principles which enable reasonable and morally appropriate decisions in the face of uncertainty. I will elaborate on these points now.

Let’s focus immediately on AI sentience, since it is commonly thought to be even more difficult to detect than animal sentience and its very possibility is contested. To be clear, I do not suggest that we currently should have high confidence in any view which makes claims about which AI systems would be conscious. However, I also do not think that we are in total uncertainty, i.e., having a degree of belief of 50% regarding the sentience of each animal or AI system is not appropriate. There are some reasons to think that some beings are more likely sentient than others.

Furthermore, there is some progress. Even though scientists and philosophers started only recently to systematically reflect on procedures to detect AI consciousness, there are already several ideas of potential empirical tests (Elamrani & Yampolskiy, 2019; Schneider, 2019). Since the need for tests of AI consciousness is getting increased attention, I expect that science will continue to make progress on validating such tests. Principled skepticism of developing valid tests of AI consciousness does not seem attractive, since there are compelling proposals for tests of animal consciousness which could even be applied to evolutionary distant species (Ben-Haim et al., 2021; Birch, 2022b; Butlin, 2020; Crook, 2021). While the AI case is more challenging, there seems to be no principled epistemic difference between testing for animal and for AI consciousness.

Arguably even more importantly, we have theoretical knowledge about consciousness we can apply to AI systems. Different AI systems are better or worse candidates for satisfying the conditions for the possession of consciousness posited by different theories of consciousness. One relatively uncontroversial upshot of a survey of different influential theories of consciousness is that metacognition^15^ and the capacity to integrate information from diverse, distributed sources are hints of sentience (Dehaene et al., 2017).^16^ In addition, we have some reason to believe that sentience correlates with domain-general and robust intelligence (Shevlin, 2020b).^17^

All this is not to say that we have already solved the problem of measuring AI sentience. If one uses the term ‘know’ in a sense which is at least minimally demanding, we don’t know which AI systems (if any) would be sentient. Nevertheless, we are also not *entirely* ignorant. When we form degrees of belief regarding sentience in different (potential) AI systems, we can justify having a higher degree of belief in sentience of some particular AI than another one. To adduce a clear example, we have more reason to think that a hypothetical generally intelligent flexible language-using AI of the year 2100 would be conscious than a chess-playing AI from 1990. AI systems which pass certain proposed tests of consciousness, have capacities for metacognition and massive integration of information and, most importantly, manifest general intelligence are significantly more likely to be sentient than AI systems which lack these properties. Hence, despite profound uncertainty, we can make some educated guesses on the sentience of various particular AI systems.

There are two canonical ways to deal with empirical uncertainty in decision making which can be applied to the case of AI consciousness. The first is to rely on precautionary principles (e.g., Birch, 2017; Browning & Veit, 2020). According to Birch (2017), we should include animals within the scope of animal protection legislation when they belong to an order for which there is sufficient evidence that at least one credible indicator of sentience is present in some species within this order. A similar principle could be used to determine which AI systems should fall within the scope of legislation which attributes basic rights to them. For instance, AI systems which possess general, flexible and robust intelligence should be protected. The same goes for systems whose behavior derives from processes of global information integration which rival mammalian brains in their complexity and functional organization. While both kinds of AI systems might frequently not be sentient, this evidence is arguably sufficient to give them the benefit of the doubt.

According to the second approach, our responsibilities to animals and AI systems are proportional to their probability of sentience (Chan, 2011; Shriver, 2020). For instance, if we have a degree of belief of 50% that a given AI is sentient, we ought—ceteris paribus—to weigh its suffering or violations of the rights it has if it turns out to be sentient half as much as suffering or violations of rights which harm beings whose sentience we are certain of. It follows that we have some responsibilities to a large group of potential future AI systems and animals although we have stronger obligations in respect to members of this group which have a higher probability of sentience. This approach solves the problem of recognizing one’s moral obligations in the face of uncertainty about PMP. To quote Chan (2011, p. 340): “From this persistent uncertainty of mental phenomena comes certainty of responsibilities to non-human organisms, although these responsibilities are discounted by the uncertainty.”

Both approaches are complementary. We should use a precautionary approach to determine the set of beings whose interests we should in general include in protective legislation and our moral deliberation. A principle analogous to the one suggested by Birch is ideally suited to make this set large enough that it hopefully does not exclude any sentient beings but sufficiently small that it is feasible in legal and moral practice. Then, if individuals or institutions consider decisions which might affect the interests of beings contained within this set, they should weigh their interests proportionally to their probability of sentience, as suggested by Chan.^18^

To conclude, even if sentience is the only viable criterion of PMP, we are not ignorant of our moral obligations towards non-human beings. While uncertainty of sentience is indeed profound, we are able to make some non-arbitrary, tentative assessments of the (subjective) probability of sentience for different AI systems. This allows us to formulate moral demands which respect and are geared towards the uncertainty that persists in respect to the distribution of PMP.

## Conclusion

In this paper, I have shown that the role of sentience in assessing which beings have moral status is independent of our prospects for discovering the distribution of sentience. If the arguments which suggest that sentience determines psychological moral patiency are sound, then the search for PMP is necessarily equivalent to the search for sentience. Hence, doubts about the epistemic accessibility of sentience cannot support independent criteria for ascribing PMP. If it turns out that the distribution of sentience cannot be known, then the distribution of PMP cannot be known either. However, the inescapability of sentience as a criterion of PMP does not constitute an insurmountable problem for ethics. As I have argued, we are not entirely clueless regarding the distribution of sentience and we are subject to determinate and epistemically accessible moral obligations that are sensitive to our uncertainty about sentience.

## Data Availability

Not applicable.

## Footnotes

1 The term ‘moral patiency’ is also sometimes used to refer to the same property as ‘moral status’.

2 That being said, the possession of moral status by a being may be only necessary, not sufficient, for the existence of obligations to that being. One may think that one can be in the wrong relationship to a being to possess obligations to it, even if other people might.

3 Consequently, even if entities without a mind, like an ecosystem, have moral status, they do not have PMP.

4 Examples are views holding that we have intrinsic moral obligations to the environment, sacred buildings or our ancestors.

5 While widely shared, the standard view is not without critics. In particular, some authors question whether sentience is necessary for moral status (e.g., Humphreys, 2016; Kagan, 2019). Beyond its narrowly ethical role, sentience is also often regarded as an arbiter of whether animals deserve legal protection (Birch, 2017; Browning & Veit, 2022; Cochrane, 2018).

6 For an argument that non-valenced experiences ground moral status too, see Chalmers (2022).

7 This reason can be overridden by stronger contrary reasons.

8 According to one interpretation (Shevlin 2020a), the literature on animal pain frequently evades explicit mention of consciousness (e.g., Sneddon et al., 2014) due to its perceived intractability. If so, then they subscribe to the epistemic objection and propose pain or negative affective states more generally as an alternative criterion of PMP. I thank an anonymous reviewer for pointing out that Coeckelbergh (2009) makes an epistemic argument similar to the ones presented here in the context of the debate on the distribution of moral agency.

9 There are also reactions to the epistemic inaccessibility of sentience which do not rely on proposing competing criteria of PMP, e.g., based on precautionary principles (Birch 2017). This will be further discussed in section “Ethics and Uncertainty of Sentience”.

10 For a discussion of epistemic limits in relation to the definition of animal welfare, see also Birch (2022a).

11 This is not, strictly speaking, correct, since sentience may be necessary or sufficient for PMP—just not both—even if sentientism is false. However, this does not relevantly change the subsequent argument. To modify the argument’s lesson: The *extent* to which sentience can be used as criterion of PMP depends on the extent to which sentience grounds PMP, i.e., whether sentience grounds it partially, whether there are additional grounds of PMP etc. It nevertheless follows from my argument that epistemic concerns are irrelevant to the choice of criteria of PMP.

12 The analogy even oversells the relevance of the knowability of sentience to the justification of sentientism. In the case of water, we need to test the chemical structure of some samples to infer that water is grounded in H2O. In the case of sentientism, we mainly do not proceed via identifying specific psychological moral patients and examining whether they are sentient. Instead, we use the method based on thought-experiments sketched in the preceding paragraph.

13 F stands in for preference satisfaction, behavioral equivalence, cognitive equivalence, the possession of affective states and other suggestions of alternative criteria of PMP that were already mentioned.

14 In the justification of his criterion of cognitive equivalence, Shevlin (2021) also points to uncertainty about the grounds of PMP as a reason to look for an ecumenical criterion which is compatible with different views on the grounds of moral status. I agree that uncertainty on whether sentience or some other property determines PMP speaks in favor of criteria of PMP which are not just referring to sentience. Whether reasonable doubts about sentience as ground of PMP really exist is a question belonging to normative ethics which is beyond the scope of this paper. I note, however, that this argument is in tension with the epistemic objection. An ecumenical criterion needs to be based on a property F which tracks sentience as well as other putative grounds of PMP (e.g., agency or reason). Thus, it presupposes that we have a viable indicator of sentience at our disposal.

15 I understand metacognition—as is standard—as the capacity to form representations of one’s own cognitive states.

16 The evidential connection between metacognition, widespread bi-directional information integration or both on the one hand and sentience on the other is suggested by, among others, global-workspace theory, integrated-information theory, recurrent-processing theory and higher-order theory.

17 Due to constraints of space, I can only allude to some elements of the vast literature on non-human sentience.

18 As remarked by Chan (2011, p. 335), there may arise cases where systems are so alien to us that we have no idea what might benefit or hurt them. In those cases, we are permitted to ignore their interests.
